# Diagnostic Yield of Upper-Tract Imaging in Visible Haematuria: A Single-Centre Retrospective Clinical Audit

**DOI:** 10.7759/cureus.99915

**Published:** 2025-12-23

**Authors:** Simone Sim, Shahzad Ahmad

**Affiliations:** 1 Department of Urology, Epsom and St Helier University Hospitals, London, GBR

**Keywords:** ct urogram, environmental sustainability, gross haematuria, heamaturia one stop clinic, upper tract urothelial carcinoma

## Abstract

Introduction

Visible haematuria (VH) is a common presenting symptom of urological malignancy, yet the optimal upper-tract imaging modality remains debated. Recent Getting It Right First Time (GIRFT) Greener Pathway guidance recommends ultrasound as the first-line investigation, reserving CT urogram (CTU) for high-risk patients. Our District General Hospital (DGH) currently performs CTU for all VH patients, regardless of risk factors, with adequate renal function. This audit aimed to evaluate the diagnostic yield of upper-tract imaging in our local VH pathway and to review our current local haematuria protocol.

Methods

A retrospective audit was conducted at a UK DGH of all consecutive patients referred to a nurse-led Haematuria Telephone Assessment Clinic with VH between 15 June and 15 September 2025. Data on demographics, clinical factors, cystoscopy findings, and upper tract imaging results were collected from electronic medical records. Descriptive statistics were used to evaluate the diagnostic outcomes of upper tract imaging.

Results

In this cohort study, the median age was 69 years old and patients were predominantly male (75%, n=75). Out of the 91 cystoscopies performed, four patients (4.3%) had a diagnosis of bladder cancer. All 100 patients underwent upper tract imaging with potentially malignant findings detected in 18% (n=18) of patients: three renal masses, five ureteric abnormalities, nine bladder findings, and one advanced prostate cancer in known metastatic disease. Among those with ureteric findings, four patients underwent further investigations which came back negative for malignancy. The last remaining patient was deemed too frail and was managed expectantly. Other benign pathologies included urolithiasis (16%, n=16), pyelonephritis (1%, n=1), renal cysts (7%, n=7) and pelvi-ureteric junction obstruction (1%, n=1).

Conclusion

While CTU effectively identifies upper tract pathology in VH, its diagnostic yield in upper tract malignancy remains low. The use of CT needs to be balanced against its diagnostic yield, patient safety and environmental impact. Local adoption of the new GIRFT guidelines in our DGH still needs to be reviewed as formulating an adequate risk-based approach for upper tract imaging requires further discussion. Further prospective, multicentre studies are required to refine evidence-based imaging pathways for VH.

## Introduction

Visible haematuria (VH) is commonly the presenting symptom in patients with bladder and upper urinary tract urothelial cancer (UTUC), and in some instances, renal cancer [1]. The estimated incidence rate of these cancers among patients with VH is significant at about 20% [1]. Established risk factors include increasing age, male gender and smoking history [2].

Given the malignancy risk, VH is often investigated using direct visualisation of the bladder with flexible cystoscopy and imaging of the upper tracts [3]. In the United Kingdom, there was no national guidance on the choice of upper tract imaging until the most recent Getting It Right First Time (GIRFT) Greener Pathway guidelines for bladder cancer [4]. This is a national programme led by NHS England to improve the treatment and care of patients, with a particular focus on sustainability to meet the NHS commitments of delivering a net-zero health service [5]. In this guideline, it is recommended that ultrasound (US) of the urinary tract should be performed as a first-line investigation for VH instead of CT [4]. The use of CT urogram (CTU) should be reserved for patients with symptoms unexplained by cystoscopy and US, and who are deemed “high risk” based on clinical factors such as age, smoking history and persistence of symptoms [4]. This recommendation is based on the low prevalence rate of UTUC [2]. Thus, there is a low risk of missing these cancers when US is used first line instead [5].

In our local District General Hospital (DGH), the current protocol is that all patients with VH are investigated by CTU first-line regardless of risk factors, provided they have adequate renal function for contrast administration. This is defined as an estimated glomerular filtration rate (eGFR) of 40 ml/min/1.73m^2^ or more. In the event of poor renal function, US is performed instead. Given the most recent guidance, the routine use of CTU for all VH patients is questioned and we aim to assess the diagnostic yield of upper tract imaging to review our current local haematuria pathways and choice of upper tract imaging modality.

## Materials and methods

Patient selection

This is a retrospective clinical audit conducted at a DGH in the United Kingdom. The study included all consecutive patients referred via the nurse-led Haematuria Telephone Assessment Clinic (TAC) over a three-month period, from 15 June to 15 September 2025. Patients eligible for inclusion were all patients referred for the investigation of visible (macroscopic) haematuria. Exclusion criteria were patients with non-visible (microscopic) haematuria, a history of bladder or UTUC, and for whom medical records were unavailable.

Diagnostic work-up and local pathways

The Haematuria TAC was set up to triage patients referred under the haematuria two-week-rule pathway to streamline investigations for urinary tract cancers. Most of the referrals are made by primary care physicians and general practitioners, but internal referrals from other subspecialties are also reflected in this clinic. For patients with VH, patients are counselled about a flexible cystoscopy and are booked for a CTU or US urinary tract depending on their renal function. There are instances where prior imaging has already been performed either by the referring team/clinician or for a separate indication and a urologist is consulted if repeat imaging is required. If a nurse-led TAC is not appropriate, the patient is booked for a face-to-face consultation with a urologist instead.

Data collection

Eligible patients were identified through the hospital booking system and electronic medical records (Cerner, Powerchart). Data were extracted manually and anonymised for analysis. The variables of interest include patient demographics, clinical factors (catheter use, previous pelvic radiotherapy, history of urinary tract infections, anticoagulant or antiplatelet use), history of previous investigations for haematuria, cystoscopy findings and upper tract imaging findings. Data was collected and analysed on Microsoft Excel (Microsoft Corporation, Redmond, USA). Descriptive statistics were used to summarise demographic data and diagnostic yield on imaging.

Ethical considerations

Given the retrospective nature of the study, formal ethical approval was not required under local research governance guidelines. The study was registered and approved as a clinical audit with the hospital governance team (audit reference: Uro2526-5).

## Results

A hundred patients were identified over a three-month period who were referred to the Haematuria TAC from 15 June to 15 September for VH.

Patient demographics and clinical factors

The median age is 69 years (range: 21-89). The majority of the patients were male (n=75, 75%). Regarding smoking status, 12% (n=12) were current smokers, 33% (n=33) were ex-smokers and 55% (n=55) never smoked. Only two (2%) patients had a family history of urothelial cancer; 19% (n=19) of the patients had previous investigations for VH, which were negative for any cancer. Investigations would be in the form of a cystoscopy of any kind (rigid or flexible) and upper tract imaging. Fifteen (n=15) patients had VH associated with urinary tract infection, which was confirmed with a positive urine culture and microscopy along with symptoms of dysuria. Three (3%) patients had a long-term catheter in situ, and 12 (12%) patients had a history of radiotherapy, all of which were performed due to prostate cancer; 33% (n=33) of patients were on anticoagulant or antiplatelet therapy at the time of presentation (Table 1).

**Table 1:** Patient demographics UTI: Urinary tract infection

Demographic	Cohort N = 100 (%)
Median age in years (range)	69 (21-89)
Gender	
Female	25 (25)
Male	75 (75)
Smoking Status	
Current	12 (12)
Ex-smokers	33 (33)
Non-smokers, never smoked	55 (55)
Family History of Urothelial Cancer	2 (2)
Previous Investigations for Haematuria	
Previously benign	19 (19)
No previous investigations	81 (81)
History of concurrent UTI	
Single episode UTI	11 (11)
Recurrent UTI	4 (4)
No UTI	85 (85)
Long-term catheter in situ	3 (3)
Previous pelvic radiotherapy	12 (12)
Anticoagulation use	
Warfarin	2 (2)
Direct oral anticoagulant (DOAC)	18 (18)
Antiplatelet use	
Single	7 (7)
Dual	6 (6)

Cystoscopy findings

Out of the 91 cytoscopies performed, the majority (n=84, 92%) were negative for findings of tumours or lesions. Four (4.3%) patients had tumours with typical papillary appearance for urothelial bladder cancer and were booked for resection surgery. Three (3.2%) patients had raised erythematous areas that were subsequently biopsied and diathermied under general anaesthetic. Nine cystoscopies were not performed for the reasons listed in Table 2.

**Table 2:** Reasons for not having a cystoscopy on referral

Reason	N
Other obvious causes of haematuria on CT not needing flexible cystoscopy	4
Recent cystoscopy/did not need repeating	2
Patient declined or did not attend	2
Patient requested transfer of care to private practice	1

Upper tract imaging findings

**Figure 1:**
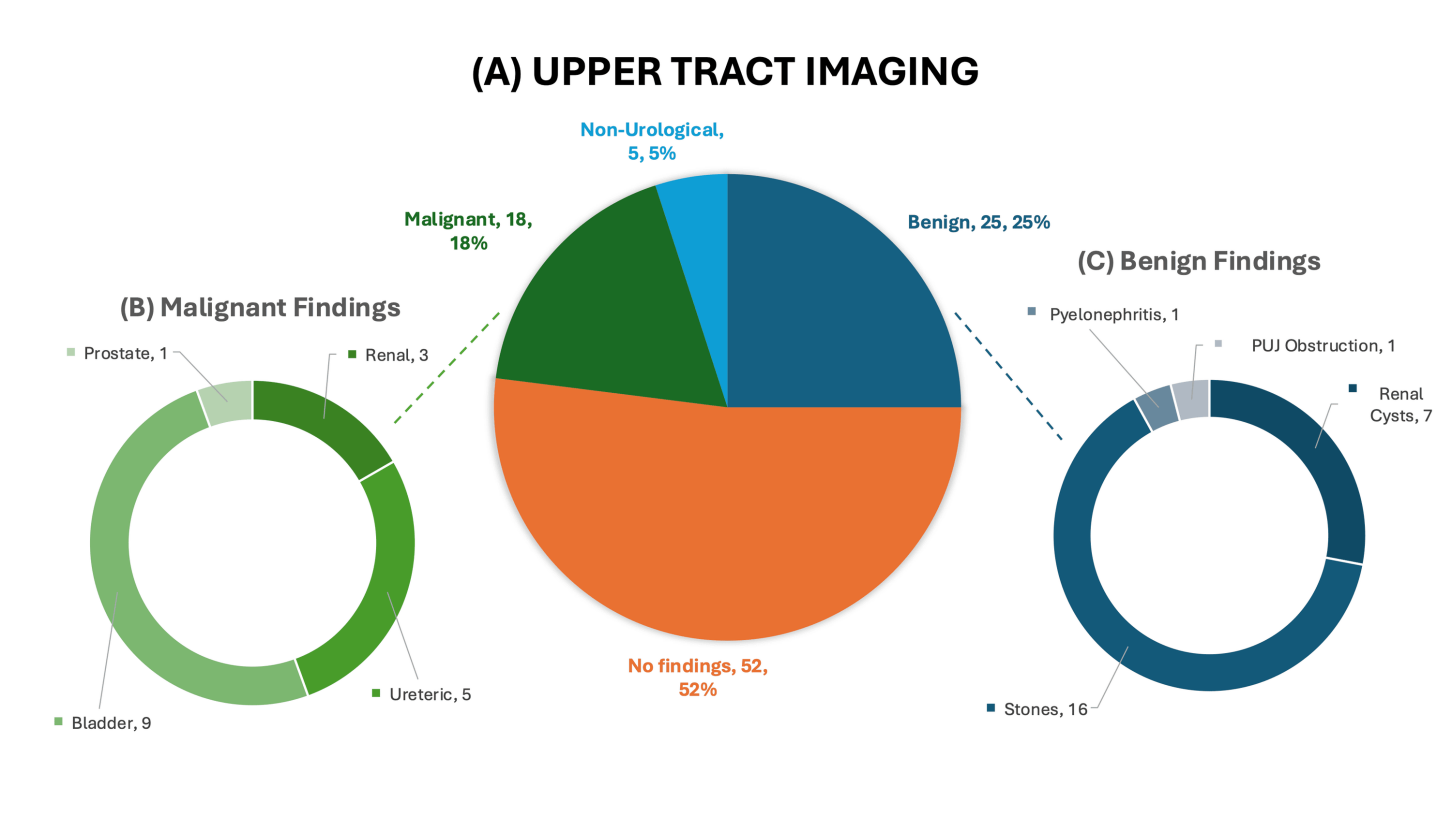
Three pie charts labelled A, B and C showing the findings on upper tract imaging. Pie chart A represents the overall categories of findings. Pie chart B represents the origin of malignant findings and pie chart (C) represents the causes of benign findings PUJ: Pelvi-ureteric junction

All patients underwent imaging of their upper urinary tract. The majority (94%, n=94) had a triple-phase CTU, while two patients had a non-contrast CT and four patients underwent US imaging only; 83% (n=5) of those who did not have a CTU had imaging performed by the referring clinician and were deemed sufficient on review by a urologist. One patient had an US instead of CTU due to their poor renal function. Overall, potentially malignant findings were seen in 18% (n=18), benign urological findings in 25% (n=25), non-urological incidental findings in 5% (n=5) and no findings were seen in 52% (n=52) (Figure 1A).

Regarding potentially malignant or suspicious findings, these include three renal masses, five ureteric abnormalities, nine bladder findings, one case of metastatic prostate cancer in a patient with known disseminated disease (Figure 1B). Of the renal lesions, two were large (>10cm) tumours and were referred for urgent nephrectomy, while one small mass was managed with a watchful-waiting approach following multidisciplinary team review. Among the ureteric abnormalities, four patients (80%) underwent further investigations and review, all of which were negative for malignancy. The remaining patient was deemed too frail for invasive assessment and was managed expectantly. Of the nine bladder abnormalities, five were discrete masses that had urgent resection surgery and the remaining four were described as bladder wall thickening only; 50% (n=2) of them had corresponding erythematous carpet-like changes that had cystobiopsy under general anaesthetic, 25% (n=1) had cystitis-like findings on cystoscopy and the remaining patient requested transfer of care to a private practice. 

Regarding benign pathology (Figure 1C), urolithiasis represented the largest subgroup, identified in 16 patients (16%). This included ureteric stones (n=4, 25%), renal stones (n=6, 37%) and bladder stones (n=5, 31%) with one patient (6%) demonstrating both renal and bladder calculi. Most cases (n=10, 62.5%) were managed conservatively in the first instance, while the remaining were scheduled for intervention in the form of lithotripsy, ureteroscopy or cystolitholapaxy. Other urological findings include a known pelvi-ureteric junction obstruction (n=1, 1%), renal cysts that needed interval imaging for surveillance (n=2, 2%), simple renal cysts (n=5, 5%) and simple pyelonephritis (n=1, 1%). Other non-urological incidental findings (Figure 1A) were liver cysts (n=3, 3%) and gynaecological conditions (n=2, 2%).

## Discussion

Haematuria is traditionally divided into two categories: ‘visible’, ‘macroscopic’ or ‘frank’, where it is instantly recognised, or alternatively ‘non-visible’, ‘microscopic’ or ‘dipstick’ when it is only detected on examination of the urine through urinalysis or microscopy [6]. This distinction is important as VH has a higher risk of detecting underlying malignancy compared to non-VH. The rates of cancer detection in VH are in the range of 23-39% [7-10] compared to 0-16% [7,11] in non-VH. In our study, 18% (n=18) of patients had findings concerning urinary tract cancer, with a definitive diagnosis in 10 (10%) of patients. 

Of all urinary tract malignancies, bladder cancer is the most common [2,6], and currently, direct visualisation via cystoscopy is the gold standard method for bladder cancer detection [12,13]. Therefore, the use of imaging is to detect benign and malignant causes of VH in the upper tracts. Despite the most recent recommendation by GIRFT that US is safe as first-line imaging [4], the adoption of this practice locally is still up for debate.

CT urogram vs ultrasound

The benefit of CT urography is that it offers the highest diagnostic accuracy for UTUC among all non-invasive medical imaging, with a recent meta-analysis of 1233 patients showing a pooled sensitivity of 92% and a pooled specificity of 95% [14]. UTUC typically presents on the excretory phase as a filling defect, as focal thickening of a segment of urothelial lining or as an infiltrative mass [14]. However, the magnitude of this benefit is limited given the low diagnostic yield of UTUC in VH overall, as evidenced by multiple studies [2,7,15]. Contemporary data from 44 studies and 22,9701 patients demonstrated a pooled incidence rate of 0.75% [1]. Likewise, in our retrospective audit, the UTUC rate is low at 1% (n=1). In addition, the European Association of Urology (EAU) guidance also questions the need for a baseline CTU to detect synchronous UTUC in patients with bladder urothelial cancer due to the low incidence of significant findings [16]. Overall, despite its accuracy, this makes it difficult to justify the routine use of CTU for all VH patients.

A CTU also comprises a non-contrast phase, where it can identify other benign causes of VH, such as urinary tract calculi. This is pertinent as stones are the most common benign cause in our cohort. It accounted for 16% (n=16) of cases in this study and in other contemporary data, this can be as high as 25% [14]. US is limited in this regard due to its low sensitivity in detecting urinary tract calculi, and it cannot offer accurate stone size and volume to guide treatment [17].

Despite its value in diagnostic accuracy, CT urography has its disadvantages. This includes increased exposure to radiation, the need for iodinated contrast and its environmental impact. The three phases of a CT urography carry potential for substantial radiation exposure to the patient [18]. The use of iodinated contrast, though generally safe, also puts patients at risk of side effects such as hypersensitivity reactions, contrast-induced nephropathy and contrast-induced thyroid dysfunction [19]. In addition, as sustainability is a growing priority when delivering healthcare services, using US instead of CT urography will reduce carbon emissions substantially. It is estimated to reduce carbon emissions by 9.9kg CO2e per patient, which translates to 32 tonnes CO2e across England annually [4].

In contrast, using US as first-line imaging has the benefit of avoiding radiation, avoiding iodinated contrast and reducing carbon emissions and costs [4,20]. The use of US has proven to require less energy consumption (both when in use and during idle time) and produces less carbon emissions [21]. It overall has a more eco-friendly life-cycle when considering manufacturing, transportation and disposal [21]. Using US can also aid in the development of a true one-stop clinic; patients would not need separate hospital appointments to complete the investigation of VH, which would have further financial and environmental gains [4].

A risk stratification approach

Ultimately, a risk-stratification approach is needed when imaging the upper urinary tract for VH. The IDENTIFY trial has developed a risk calculator [22] which evaluates the patient’s age, type of haematuria, smoking history, and other clinical factors to quantify their risk of urothelial cancer [2]. Although it does give an estimated risk of detection of urothelial cancer, it does not offer guidance as to when a CTU is indicated over a US, and the decision is left to local protocols and the discretion of the clinician. In our study, the five patients who had concerning ureteric findings on CT urography had an IDENTIFY risk of 18.5% to 42.6%, with all (n=4) of the patients fit for further investigations having reassuring, negative results. Additionally, the Royal College of Radiologists have recommended that all patients under the age of 40 presenting with VH (where urinary stones, infection or trauma are not suspected) should have US as their initial imaging [23] due to the low risk of urological cancer and their increased risk of radiation-induced cancers. These findings underscore the importance of individualised imaging pathways to safely rationalise CT urography use, particularly in younger or lower-risk patients, without compromising diagnostic accuracy or patient safety. 

Limitations

This study has several limitations. Firstly, this was conducted as a single District General Hospital, which limits the generalizability of findings to other centres, with different patient demographics, referral patterns and haematuria protocols. The sample size, while representative of local practice, is insufficient to guide wider practice. The absence of long-term follow-up also limits the ability to assess delayed cancer diagnosis if patients face recurrent VH. Finally, as the study did not directly compare CT urography with US within the same patient cohort, its capacity to draw definitive conclusions about the relative diagnostic yield of each modality is limited. Despite these, the findings are still reflective of contemporary, multi-centre studies. It also highlights the need for future prospective, randomised studies to further validate risk-based imaging strategies and establish evidence-based criteria for selecting the optimal imaging modality in VH, given the new GIRFT guidance.

## Conclusions

This retrospective audit highlights the value of CTU as a comprehensive means of detecting benign and malignant causes of VH. However, it also reiterates the low detection rate of upper tract malignancy; thus, the use of CT needs to be balanced against its diagnostic yield, patient safety and environmental impact. Overall, local adoption of the new GIRFT in our DGH still needs to be reviewed as formulating an adequate risk-based approach requires further discussion. Further multi-centre, randomised studies are needed to define and validate imaging pathways for VH.
